# Surgical Stabilization of a Cervical Fracture in a Patient with Ankylosing Spondylitis in the Sitting Position

**DOI:** 10.7759/cureus.6625

**Published:** 2020-01-10

**Authors:** Nathan A Shlobin, Nader S Dahdaleh

**Affiliations:** 1 Department of Neurological Surgery, Feinberg School of Medicine, Northwestern University, Chicago, USA; 2 Department of Neurological Surgery, Feinberg School of Medicine Northwestern University, Chicago, USA

**Keywords:** ankylosing spondylitis, cervical spine, combined anterior-posterior approach, posterior long segment fusion, seated, sitting, unstable fracture

## Abstract

Ankylosing spondylitis is a seronegative spondyloarthropathy promoting alteration of the integrity and biomechanics of the spine. This leads to a brittle and hyperkyphotic spine with an increased risk of cervical spine fracture. Cervical spine fractures in people with ankylosing spondylitis are often unstable three-column extension injuries that are managed with posterior long segment fusions. Kyphotic deformity, body habitus, and increased airway pressures complicate these procedures. A 65-year-old man presented with neck pain following a fall from a roof. He was found to have a displaced transverse three-column fracture of C6/7. The original plan was to perform a staged circumferential cervical fusion with anterior cervical fusion first to make prone positioning for posterior fusion safer. CT after anterior cervical fusion from C5 to C7 demonstrated improved alignment of the fractured posterior elements. Due to concern of increased peak airway pressures and awkward positioning, planned prone positioning for posterior approach was abandoned. A posterior long segment fusion from C4 to T3 was performed in the seated position. CT demonstrated the hardware was appropriately placed. The patient’s hospital course was uncomplicated, and he was followed up with cervical spine x-rays. Two years later, he denied neck pain or functional impairment and x-ray demonstrated healing of the fracture. Utilizing the sitting position for the posterior cervicothoracic fusion portion of a combined anterior-posterior approach can overcome complication-spurring positioning difficulties and provide proper surgical management of an unstable cervical spine fracture in a patient with ankylosing spondylitis.

## Introduction

Ankylosing spondylitis (AS) is seronegative spondyloarthropathy involving rheumatism in the vertebral column and sacroiliac joints [1]. AS is a slow but progressive chronic disease that usually begins around age 25 [1]. Persistent systemic inflammation alters the integrity and biomechanics of the spine through bone resorption and remodeling, including multisegmented vertebral joint autofusion, ligamentous ossification, osteoporosis, and kyphosis [1,2]. These changes precipitate a brittle and rigid hyperkyphotic spine with a fourfold increased risk of spinal fracture, particularly in older patients [3]. Cervical spine fractures are most common, followed by thoracic, lumbar, and vertebral fractures [2,4]. Spinal fractures are associated with a 20% risk of spinal cord injury, with risk greatest for cervical spine fractures [4]. Fractures often necessitate surgical intervention, but surgical planning and execution is oftentimes challenging due to instability of the fracture, spinal deformity from AS, and presence of comorbidities [3-5].

Cervical spinal fractures in patients with AS most commonly include unstable three-column extension type injuries [4]. These are managed with posterior long segment fusions, involving application of multiple points of fixation in order to provide adequate biomechanical stability to combat long lever arms that create large moments about the fractured vertebra [6]. Alternatively, circumferential fusions have further augmented implant stability and made posterior fusions safer during the process of prone positioning by providing anterior column support [2].

Often preexisting kyphotic deformity in patients with AS and decreased lung capacities may portend increased airway pressures during prone positioning necessitating, in severe circumstances, surgical abortion [7]. Preexisting cervicothoracic kyphosis and body habitus may increase risk of spinal cord injury during the prone positioning process and can be ergonomically challenging during surgical stabilization [8].

We report a case of a patient with AS and severe upper thoracic kyphosis who presented with an unstable cervical three-column extension type fracture. He was managed with circumferential fusion. The posterior long segment fusion was successfully approached in the sitting position to avoid the possible complications of prone positioning.

## Case presentation

**Figure 1:**
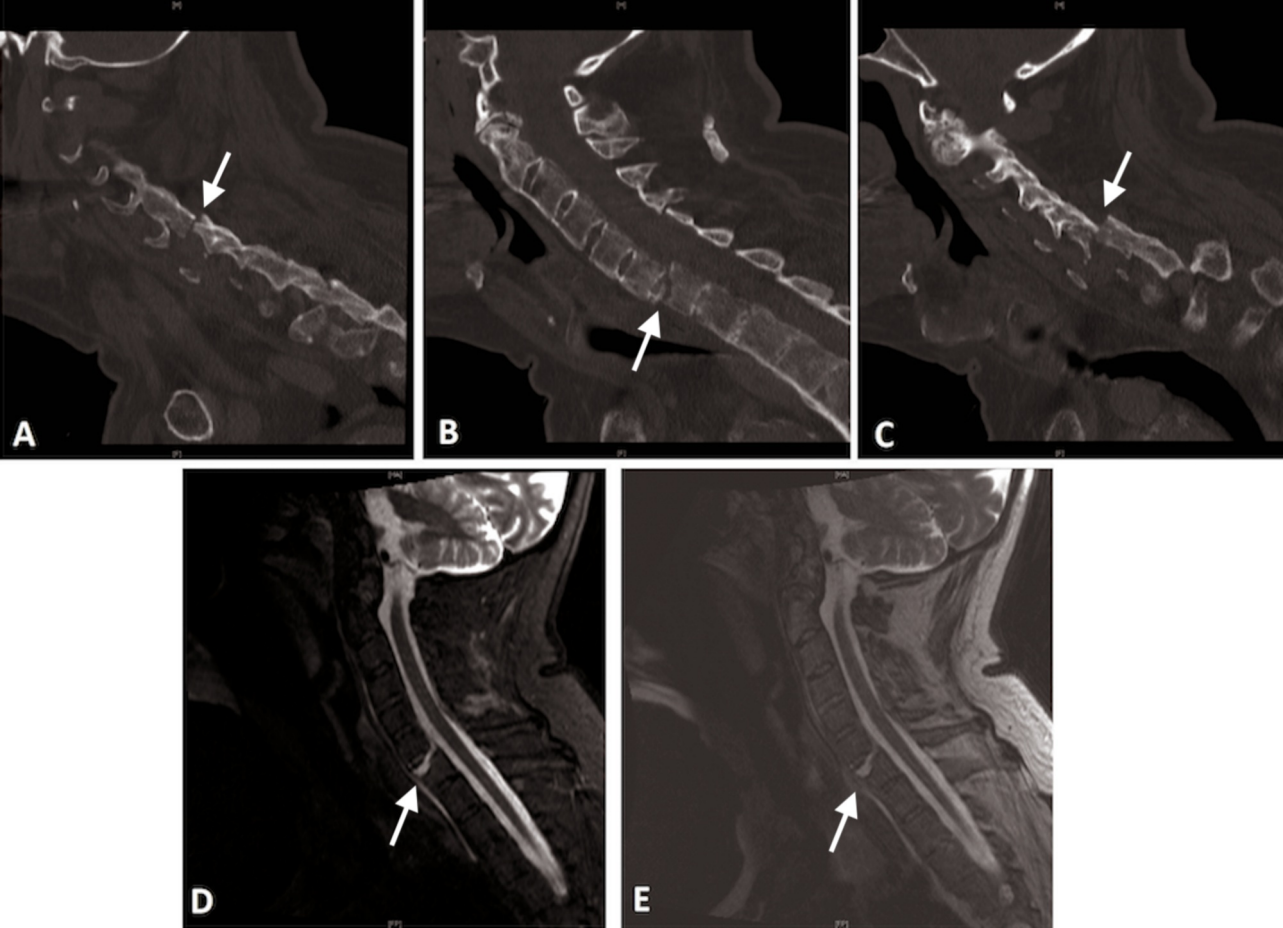
Preoperative CT scan of cervical spine CT scan of the cervical spine demonstrating a three-column extension fracture at C6/7 in the setting of ankylosis of the levels above and below. Note the three-column involvement with fracture through the ossified disk (B) and involvement and displacement of the facets (A and C). Short tau inversion recovery (D) and T2-weighted (E) sequences demonstrating increased signal segmentally at the level of the fracture indicate total segmental disruption.

A 65-year-old man with AS presented with neck pain after fall from a roof. Complete precautions were followed. He was placed in a rigid collar. He was neurologically normal after falling. A non-contrasted computed tomography (CT) revealed a displaced transverse three-column fracture of C6/7 in the setting of severe kyphosis and evidence of multilevel ankylosis consistent with AS (Figure 1A-1C). MRI confirmed these findings with disruption of the posterior elements (Figure 1D, 1E). During the process of obtaining imaging, care was taken to keep the head and neck bolstered to avoid the progression of hyperextension while spinal precautions were followed.

The original surgical plan was to perform a staged circumferential cervical fusion with anterior cervical fusion first to make prone positioning for the planned posterior fusion safer.

**Figure 2:**
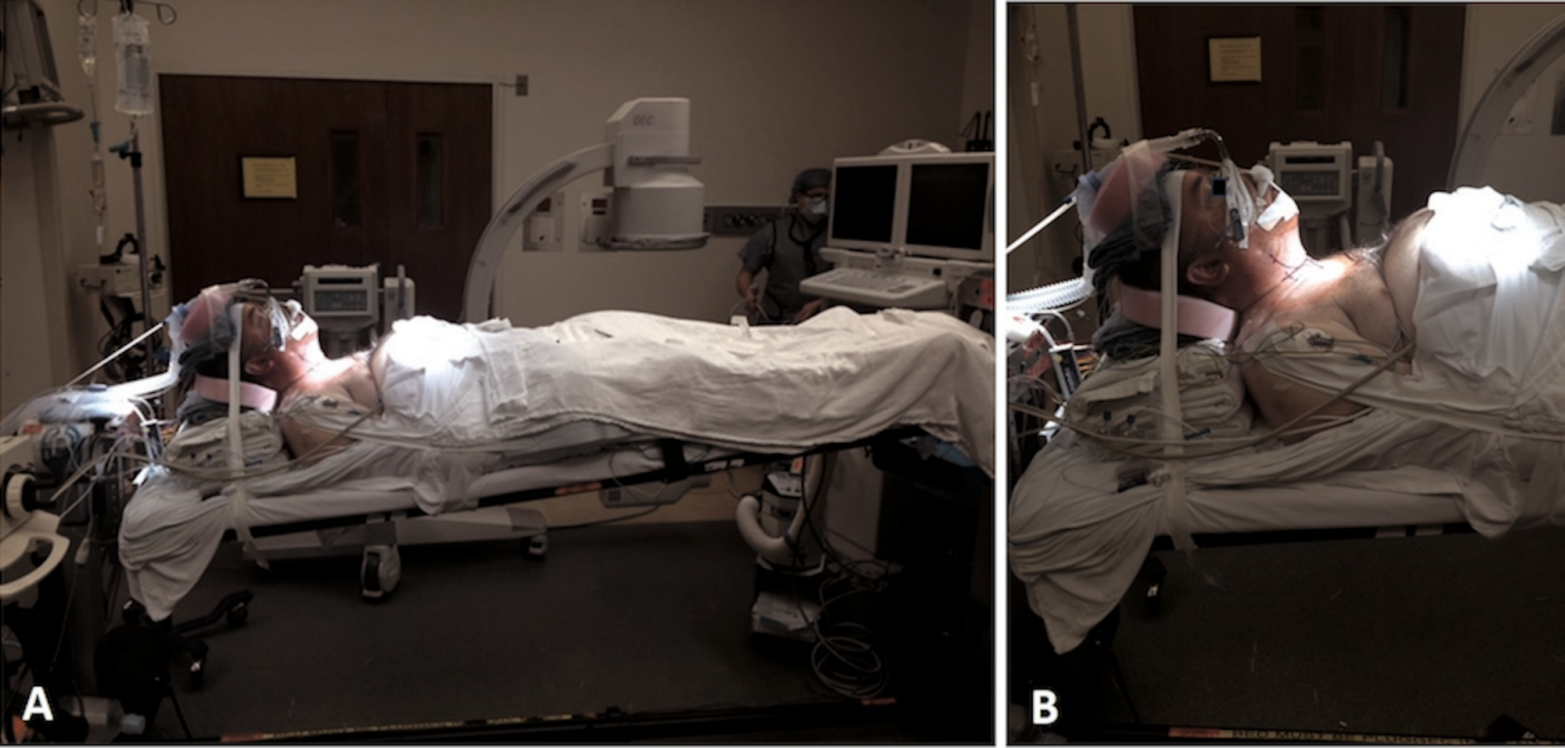
Supine positioning during anterior cervical approach Supine positioning during the anterior cervical approach necessitating use of bolsters to fill the gap between the head and the bed and a table mounted Mayfield headrest in order to support the cervical spine. This is due to the posture created by the cervical/thoracic kyphosis due to ankylosing spondylitis (A). Note elevation of the abdomen and diminished chest cavity space as the result of the body habitus due to kyphosis (B).

**Figure 3:**
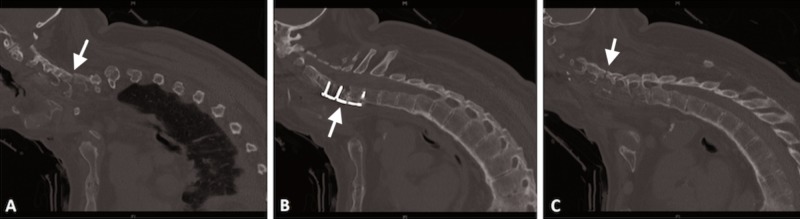
CT scan of cervical/thoracic spine after anterior cervical fusion CT scan of the cervical/thoracic spine demonstrating anterior cervical fusion C5-7 (B) with improvement of the alignment of fractured posterior elements (A, C). Note the kyphotic deformity.

The patient underwent asleep fiber optic intubation and was placed in the supine position. The patient’s head was placed in a three-point pin Mayfield headrest mounted to the table. Due to kyphosis, a posterior bolster composed of towels was placed to fill the gap between his head and neck and the operative table. Neurophysiological monitoring was used. A standard anterior cervical fusion was performed from C5 to C7 filling the gap created by the fracture with bone allograft (Figure 2). During the operation, high peak airway pressures (32-37 cm H_2_O) were noted but the procedure was completed safely. A postoperative CT showed improvement in the alignment of the fractured posterior elements (Figure 3).

**Figure 4:**
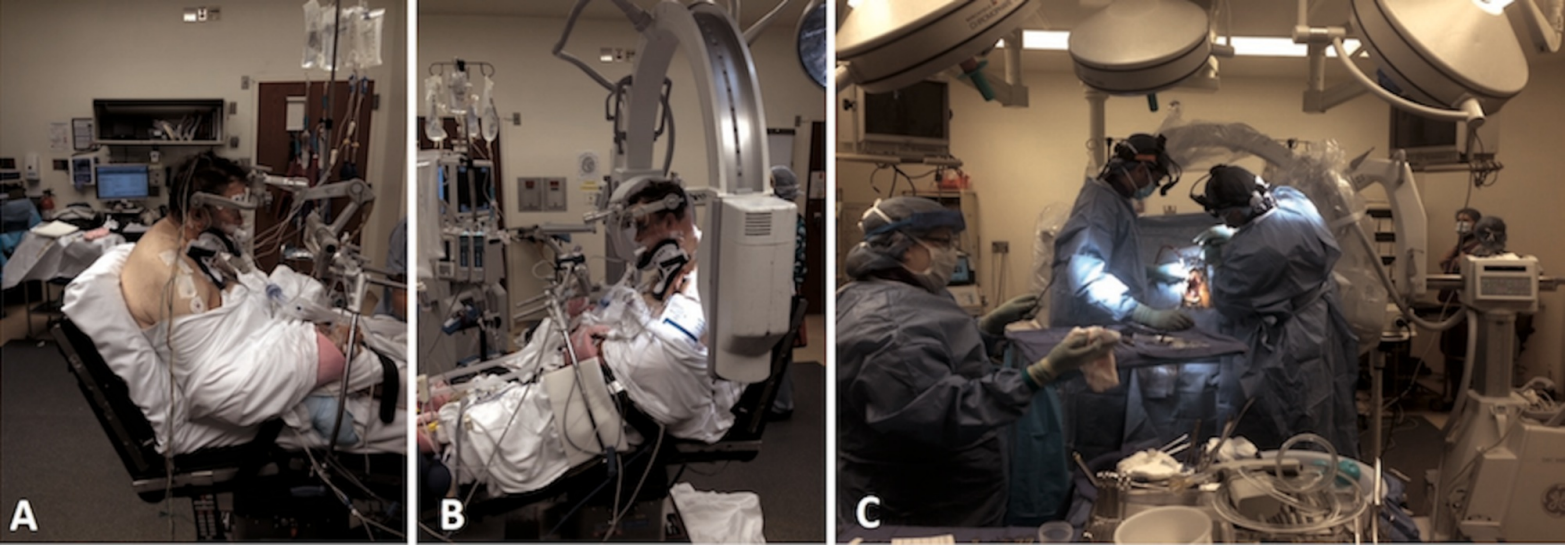
Sitting position for posterior cervical fusion Intraoperative images demonstrating the set up during the sitting position for the posterior cervical fusion (A, B). Ergonomics improved in comparison to prone positioning in the face of cervical-thoracic kyphosis (C).

Due to concern of increased peak airway pressures and awkward positioning due to kyphosis, the planned prone positioning for the posterior approach was abandoned. We performed the posterior long segment fusion in the seated position. The patient underwent asleep fiber optic intubation. A central line was placed with precordial Doppler to monitor for air embolism. Neurophysiological monitoring was used. The patient’s head was placed in a three-point pin Mayfield headrest. He was positioned in the sitting position such that his cervicothoracic kyphus was now parallel to the horizontal. Standard posterior long segment fusion spanning C4 to T3 segmentally with “free hand” placement of cervical lateral mass screws and thoracic pedicle screws was conducted. X-rays confirmed appropriate placement of screws and avoidance of pedicle violation. Corticocancellous bone allograft and demineralized bone matrix were used for posterior lateral fusion. Normal peak airway pressures were noted throughout the operation of <25 cm H_2_O (Figure 4).

**Figure 5:**
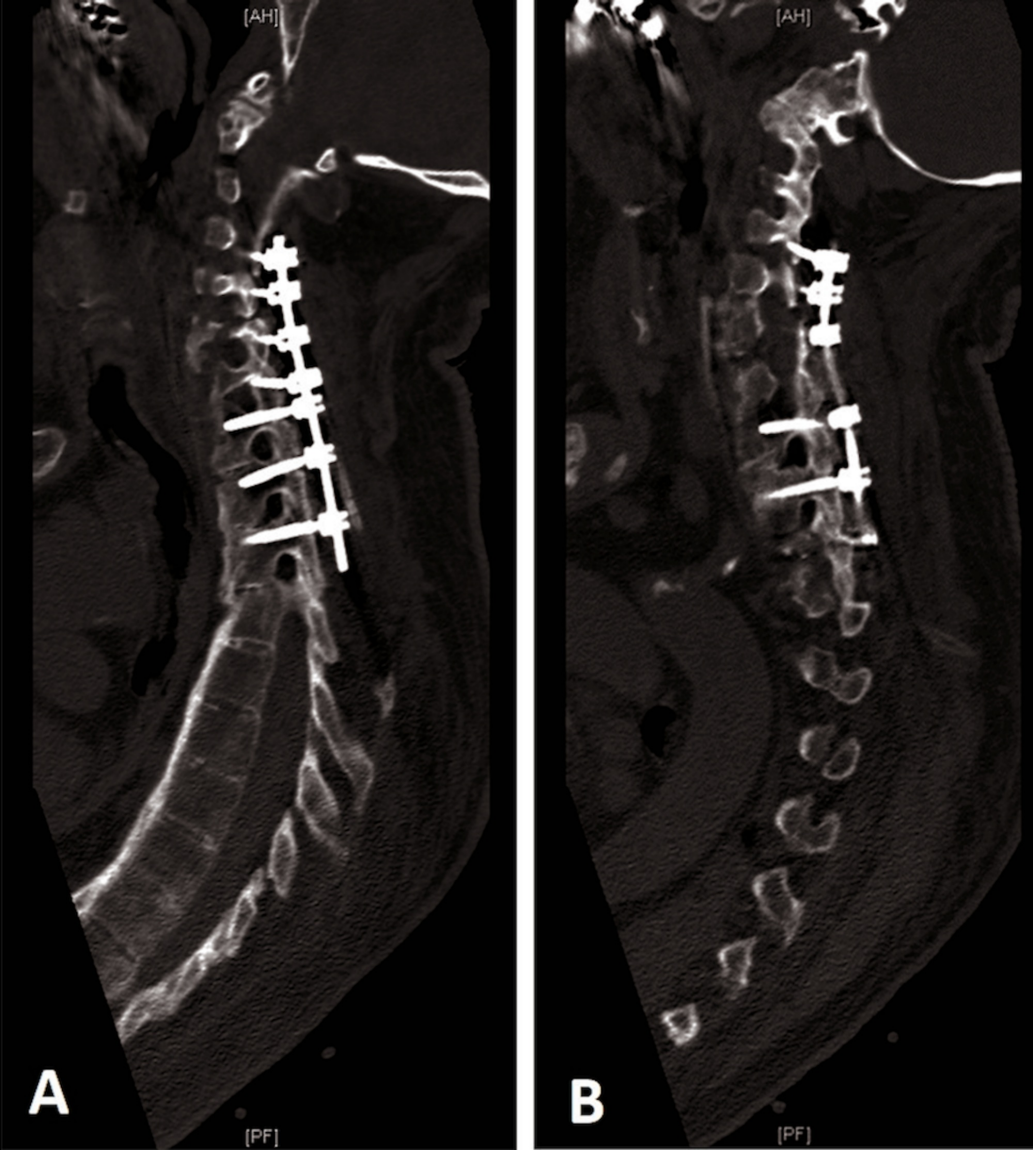
Postoperative parasagittal CT scan Postoperative parasagittal CT scan (A, B) demonstrating appropriate hardware placement spanning C4-T3 segmentally. Knowledge of the anatomy cannot be overemphasized, especially for “free-hand” screw placement in patients with ankylosing spondylitis, as normal anatomy is often obscured due to the presence of autofusion.

Postoperatively, the patient was kept in a rigid collar. His neurological examination was normal. His hospital course was smooth and uncomplicated. A postoperative CT was obtained demonstrating appropriate hardware placement (Figure 5).

**Figure 6:**
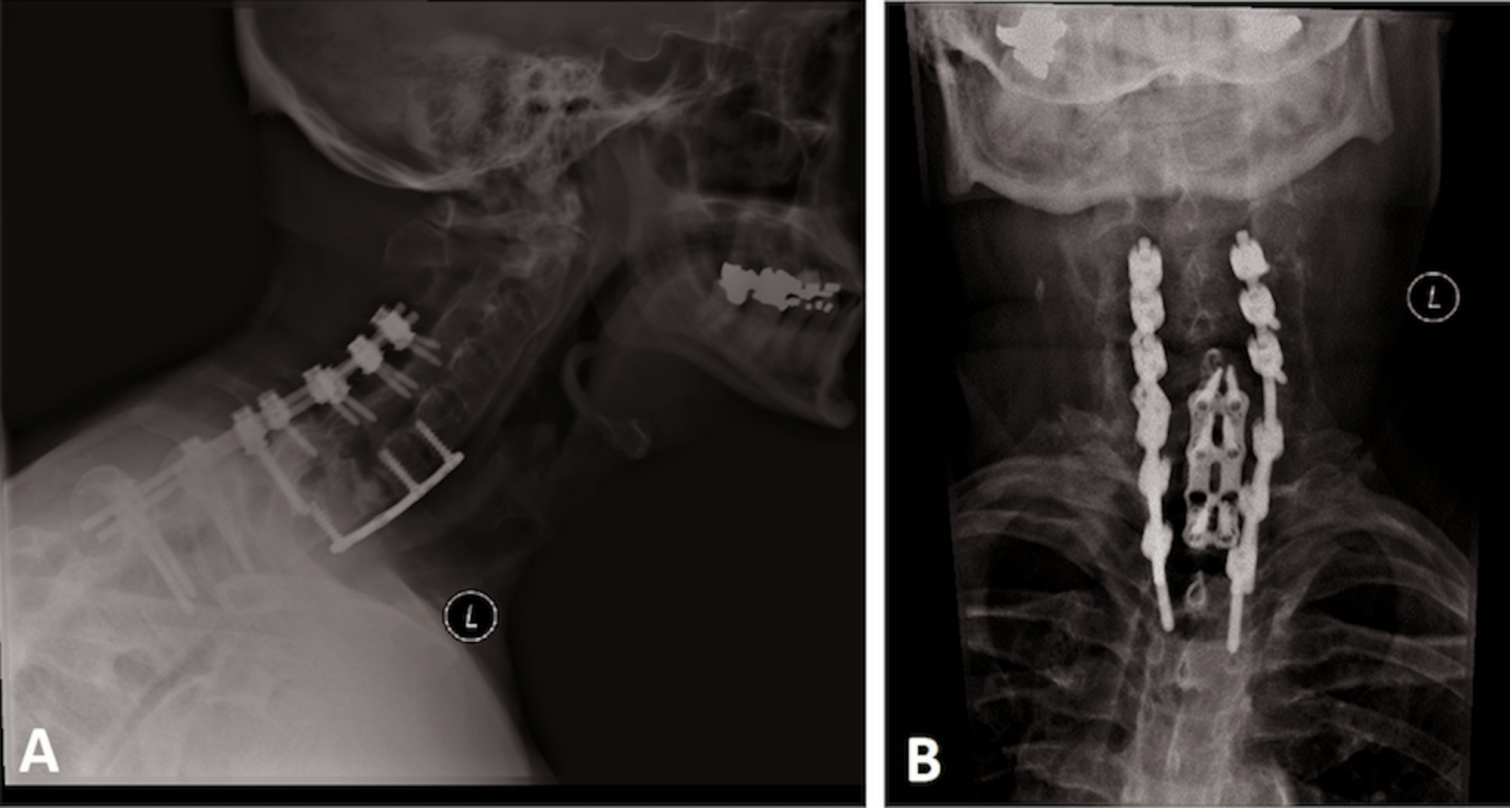
X-rays at most recent follow-up visit Lateral (A) and anteroposterior (B) x-rays of the cervical spine demonstrating healing of the fracture and appropriate hardware placement.

He followed up in clinic on regular basis with cervical spine x-rays. During his two-year follow-up visit, he denied any neck pain and was pursuing his job normally. An x-ray during his last visit was obtained demonstrating healing of the fracture (Figure 6).

IRB approval for this case report was neither required nor sought, since only de-identified data were included. Our operative consent, which the patient consented to and signed, included a statement about the possibility of intraoperative pictures taken and used for educational purposes. Intraoperative pictures concealed the patient’s eyes for privacy. 

## Discussion

Conservative management was historically considered the gold standard for treating cervical spine fractures in AS patients, consisting of bed rest, traction, and immobilization with a halo vest [9]. However, these methods have been associated with pulmonary complications, decubitis, distraction, neurologic deterioration, failed union, and worsening of kyphosis [2,9].

Surgical management of patients with AS who sustain spinal fractures improves survival and functional outcomes, such as alleviation of neurological function injury and breathing and eating difficulties [2,10,11]. However, surgery for spinal fractures associated with AS is complex and replete with risk due to multiple factors [4,5]. Patients with AS often have greater comorbidities, such as hyperkyphosis-related reduction in pulmonary function [12-14]. Increased risk of spinal cord injury due to instability of the fracture necessitates careful consideration of transfers and operative positioning [3,15,16]. Severe kyphosis limits the ability to position the patient for surgical intervention anteriorly or posteriorly [4,16].

Surgical interventions employ either a posterior or combined approach. Both approaches lead to similar outcomes and improved outcomes and lower complication rates and failure than an anterior approach [17,18]. A posterior approach, indicated if the anterior weight-bearing column is aligned properly and lacks fracture gaps, commonly involves either posterior fixation with screws and plates or rods or the addition of a posterior bone graft [9,19]. Combined anterior and posterior instrumentation is necessary when the structural integrity of the vertebral body has been compromised, and kyphosis is present at the fracture site [9]. Whether a circumferential fusion or a posterior fusion is chosen, a posterior long segment fusion construct should be achieved in the treatment plan [2]. This involves application of multiple points of fixation to provide adequate biomechanical stability to combat long lever arms that create large moments about fractured vertebra [6].

Using pedicle screws augments fixation [9]. Image-guided navigation technology and robot-assisted pedicle placement provide more accurate pedicle screw placement than conventional methods. Robot-assisted technologies have numerous benefits, such as improving ergonomics and dexterity, eliminating physiological tremor, allowing repetitive movements, holding of tools for long periods of time, and promoting three-dimensional visualization. These factors assist surgeons in the placement of screws along a defined trajectory. Image-guided navigation technology and robot-assisted methods can be incorporated to improve accuracy in spinal surgical interventions [20].

The patient’s body habitus, increased risk of ventilatory complications due to peak airway pressures, and surgical ergonomics required a posterior approach through a sitting position. This was done successfully while addressing the aforementioned concerns.

## Conclusions

The unique treatment approach for a case of unstable cervical fracture in a patient with severe kyphosis resulting from AS demonstrates that utilizing the sitting position for the posterior cervicothoracic fusion portion of a combined anterior-posterior approach can allow for proper surgical management. The sitting position overcomes complication-spurring positioning difficulties associated with the combination of fracture instability and kyphosis in AS.
